# Genome Insight and Comparative Pathogenomic Analysis of *Nesterenkonia jeotgali* Strain CD08_7 Isolated from Duodenal Mucosa of Celiac Disease Patient

**DOI:** 10.3389/fmicb.2017.00129

**Published:** 2017-02-02

**Authors:** Atul M. Chander, Ramesan G. Nair, Gurwinder Kaur, Rakesh Kochhar, Devinder K. Dhawan, Sanjay K. Bhadada, Shanmugam Mayilraj

**Affiliations:** ^1^Department of Biophysics, Panjab UniversityChandigarh, India; ^2^Department of Endocrinology, Postgraduate Institute of Medical Education and ResearchChandigarh, India; ^3^Microbial Type Culture Collection and Gene bank, CSIR-Institute of Microbial TechnologyChandigarh, India; ^4^Department of Gastroenterology, Postgraduate Institute of Medical Education and ResearchChandigarh, India

**Keywords:** genome sequencing, *Nesterenkonia jeotgali*, RAST, comparative genomics, celiac disease, pathogenicity, gut

## Abstract

Species of the genus *Nesterenkonia* have been isolated from different ecological niches, especially from saline habitats and reported as weak human pathogens causing asymptomatic bacteraemia. Here, for the first time we are reporting the genome sequence and pathogenomic analysis of a strain designated as CD08_7 isolated from the duodenal mucosa of a celiac disease patient, identified as *Nesterenkonia jeotgali*. To date, only five strains of the genus *Nesterenkonia* (*N. massiliensis* strain NP1^T^, *Nesterenkonia* sp. strain JCM 19054, *Nesterenkonia* sp. strain F and *Nesterenkonia* sp. strain AN1) have been whole genome sequenced and annotated. In the present study we have mapped and compared the virulence profile of *N. jeotgali* strain CD08_7 along with other reference genomes which showed some characteristic features that could contribute to pathogenicity. The RAST (Rapid Annotation using Subsystem Technology) based genome mining revealed more genes responsible for pathogenicity in strain CD08_7 when compared with the other four sequenced strains. The studied categories were resistance to antibiotic and toxic compounds, invasion and intracellular resistance, membrane transport, stress response, osmotic stress, oxidative stress, phages and prophages and iron acquisition. A total of 1431 protein-encoding genes were identified in the genome of strain CD08_7 among which 163 were predicted to contribute for pathogenicity. Out of 163 genes only 59 were common to other genome, which shows the higher levels of genetic richness in strain CD08_7 that may contribute to its functional versatility. This study provides a comprehensive analysis on genome of *N. jeotgali* strain CD08_7 and possibly indicates its importance as a clinical pathogen.

## Introduction

The genus *Nesterenkonia* belongs to the family *Micrococcaceae* and comprises mesophilic moderate haloalkaliphilic bacteria (Stackebrandt et al., 1995). The members of this genus stain Gram-positive having high genomic G+C content (64–72%) and are generally aerobic, catalase positive and chemo-organotrophic (Stackebrandt et al., 1995; Collins et al., 2002; Li et al., 2005). The genus is closely related to the genera *Micrococcus, Arthrobacter*, and *Kocuria* and cells are usually coccoid or rod-shaped, with or without branching, non-spore-forming and non-encapsulated (Stackebrandt et al., 1995; Govender et al., 2013). *Nesterenkonia* spp. are found ubiquitously in nature and have been isolated from various environmental niches, including extreme environments like hypersaline soils, soda lakes (Collins et al., 2002; Delgado et al., 2006), hot deserts and saline soils (Li et al., 2004, 2005, 2008). Some strains of *Nesterenkonia* spp. have been reportedly isolated from eﬄuent treatment plants of paper and cotton pulp mills (Luo et al., 2008, 2009), whereas some were even isolated from feces of AIDS patient (Edouard et al., 2014) and fermented sea food (Yoon et al., 2006). At present, 13 species of genus *Nesterenkonia* have been validly described (Aidan-Parte, 2014).

Celiac disease (CD) is an autoimmune disorder of the small intestine. Gluten is considered to be the environmental factor responsible for disease pathogenesis but gluten alone is unable to define the disease pathogenesis (Pozo-Rubio et al., 2012). It was reported that the altered intestinal microbiota composition (dysbiosis) is strongly associated with different disease presentation (Nadal et al., 2007; Wacklin et al., 2014). Dysbiosis is caused by early age infections, presence of pathobionts and intake of antibiotics (Cinova et al., 2011; Pozo-Rubio et al., 2013; Sanchez et al., 2013; Canova et al., 2014; Shmidt et al., 2014). As infections are important in CD, they need to be studied thoroughly to understand the host microbe interactions using robust genomic approaches.

Recently we reported unique microbes from CD patients and their description suggested them to be pathogens potentially involved in the disease (Chander et al., 2016a,b). In this study we report another strain designated as CD08_7, isolated from the duodenal mucosa of the small intestine and which was provisionally identified as *N. jeotgali. N. jeotgali* was initially reported to be isolated from jeotgal, a traditional Korean fermented sea food. *N*. *jeotgali* is a non-motile, slightly halophilic and Gram-positive actinomycete which grows optimally at 25–30°C in the presence of 2–5% (w/v) NaCl (Yoon et al., 2006). Although to date, there is no direct previous evidence for the presence or prevalence of *N. jeotgali* in CD patients, phylum *Actinobacteria* has been known to have a strong relation with the clinical presentations of the CD. Members of this phylum are more abundant in biopsy samples of symptomatic CD patients than in samples of asymptomatic patients (Bustos Fernandez et al., 2014). Interestingly, other *Actinobacteria* of the family *Micrococcaceae* (*Kocuria kristinae* and *Rothia mucilaginosa*) were more frequent in symptomatic patients as compared to controls whereas absent in asymptomatic patients. Thus, aided by culturomics, Sanchez et al. (2013) described the presence of *Micrococcaceae* members in CD. Members of phylum *Actinobacteria* are among the most commonly known bacteria of human duodenum. Though, genus *Nesterenkonia* has rarely been described in humans but very recently Avilés-Jiménez et al. (2016) observed a higher frequency of *Nesterenkonia* spp. in benign biliary pathology as compared to biliary tract cancer.

Whole genome sequencing (WGS) of microbes has been used to predict the functional behavior of microbes. In a similar way D’Argenio et al. (2016) not only predicted but validated the WGS predicted pathogenic role of CD gut microbes by a combination with *in vitro* approaches. They reported *Neisseria flavescens* strains as the most abundant in symptomatic CD patients. Further, WGS showed these strains possess diverse genetic composition of virulence determinant genes (particularly iron acquisition systems and hemoglobin-related genes) when compared to those isolated from control subjects. In addition, these strains isolated from patients, were able to escape the lysosomal compartment in Caco-2 cells and to activate inflammatory responses in CD patients and in *ex vivo* culture of duodenal biopsies, confirming their possible role in inflammation at the intestinal mucosa. The strain we describe here also possessed several genes for iron acquisition systems and other virulence determinant genes that may also predict its virulence capabilities.

In this study we sequenced the genome of *N. jeotgali* strain CD08_7 for the first time and attempted to map its virulence profile through a comparative genomic approach with the already published genomes of *Nesterenkonia* species *viz. N. massiliensis* strain NP1^T^, *Nesterenkonia* sp. strain JCM 19054, *Nesterenkonia* sp. strain F and *Nesterenkonia* sp. strain AN1.

## Materials and Methods

### Bacterial Strain Isolation and Identification

**FIGURE 1:**
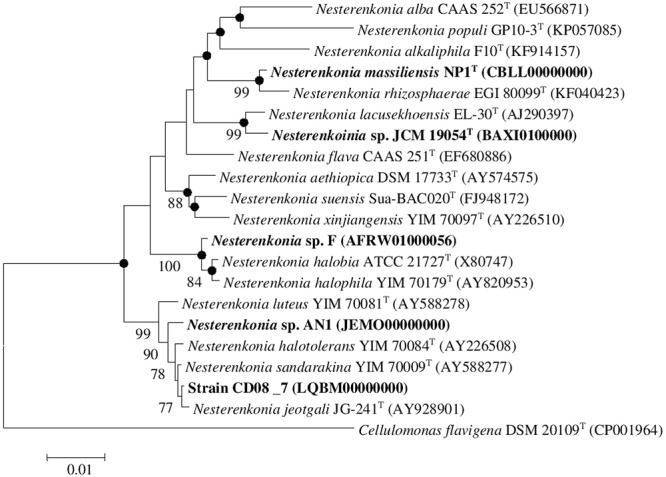
**Neighbor-joining tree.** Neighbor-joining tree based on 16S rDNA sequences, showing the phylogenetic relationship between *Nesterenkonia* species and other related members of the genus *Nesterenkonia. Cellulomonas flavigena* DSM 20109^T^ (CP001964) was used as an outgroup Bootstrap values (expressed as percentage of 100 replications) greater than 70% are given at the nodes. Filled circles indicate that corresponding nodes were also recovered in the trees generated with maximum parsimony and maximum likelihood algorithms. Bar 0.01% sequence variation.

Strain CD08_7 was isolated from the duodenal mucosa of a CD patient. The tissues samples from duodenal mucosa were recovered during endoscopy at the Postgraduate Institute of Medical Education and Research, Chandigarh, India. The samples were collected and aseptically transferred to CSIR-Institute of Microbial Technology, Chandigarh. The tissue was homogenized in sterile phosphate saline (PBS) and centrifuged at 4000 rpm for 2 min to remove debris. The supernatant was recovered and serially diluted with PBS and plated on to tryptic soy agar (TSA; HiMedia, India), incubated at 37°C in the presence of oxygen for 36 h. The individual colonies appearing in the plate were picked and plated on to fresh TSA medium which is a broad spectrum medium, further passaged for two times until separate colonies were obtained. The different pure isolates were selected on the basis of distinct cell shape and colony morphology, and these selected strains were identified by 16S rRNA gene sequencing. Antibiotic susceptibility tests for strain CD08_7 was performed by placing antibiotic disks (Icosa universal-2, HiMedia, India) on TSA plates seeded with suspensions of strain CD08_7. Characterization of the strain CD08_7 was performed according to the methods described by Kaur et al. (2016). Genomic DNA extraction and amplification was performed as previously described (Mayilraj et al., 2006). Identification of phylogenetic neighbors and the calculation of pairwise 16S rRNA gene sequence identity levels were achieved using the EzTaxon server (Kim et al., 2012) and alignments were carried out using Mega version 6.0 (Tamura et al., 2013). Phylogenetic trees were constructed using the neighbor-joining as well as maximum likelihood and maximum parsimony algorithms. Bootstrap analysis was performed to assess the confidence levels of the branching (**Figure 1**). The reference genomes of *Nesterenkonia* strains NP1^T^, F, AN1, and JCM 19054 were obtained from the NCBI genome database and bear the following accession numbers CBLL00000000, AFRW00000000, JEMO00000000, and BAXI00000000, respectively.

### Genome Sequencing and Assembly

A draft genome of strain CD08_7 was sequenced at C-CAMP^1^ next-generation genomics facility, Bengaluru, India using an Illumina HiSeq 2 × 100 platform. Library preparation was performed at C-CAMP’s genomics facility using TruSeq^®^ DNA sample preparation kit (Illumina) as per manufacturer’s instructions. About 1 μg of pure genomic DNA was sonicated using Covaris shearing to obtain 300–400 bp fragment size. The resulting fragmented DNA was cleaned up using AMPure XP beads as described by the manufacturer. Fragmented DNA was subjected to a series of enzymatic reactions that repaired frayed ends, phosphorylated fragments, and added a single nucleotide ‘A’ overhang then ligated adaptors using TruSeq^®^ DNA sequencing kit following the protocol as described by the manufacturer. Sample clean-up was done using AMPure XP beads. After ligation-clean-up, ∼300–400 bp fragments were size selected on 2% agarose with SYBR Gold gel using TAE Buffer and cleaned using MinElute column, QIAGEN. PCR amplification of adaptor-ligated fragments was done and followed by a cleanup using AMPure XP beads. The prepared libraries were quantified and then validated for quality by running an aliquot on High Sensitivity Bio analyser Chip, Agilent. Assembly was carried out with CLC Bio Workbench v7.5.1 (CLC Bio, Denmark).

### Genome Annotation and Comparative Genomics

The genome annotation for strain CD08_7 was performed using RAST (Aziz et al., 2008; Overbeek et al., 2014; Brettin et al., 2015), which is an automated genome annotation server^2^. Similarly, automated genome annotation for reference genomes of *Nesterenkonia* strains NP1^T^, F, AN1, and JCM 19054 was also accomplished using RAST. Further, the ribosomal RNA genes in the genomes were identified using RNAmmer 1.2 (Lagesen et al., 2007). The tRNA and tmRNA genes were identified by ARAGON (Laslett and Canback, 2004). Insertion sequence [IS] elements were identified by the IS finder^3^ (Siguier et al., 2006). The RAST server provides a comprehensive platform for comparing two genomes after their annotation through the seed viewer. Homology search of the protein encoding genes is carried out at protein level using Gene Locator and Interpolated Markov ModelER (GLIMMER 2) against a set of protein families called FIGfams (Aziz et al., 2008). Two genes are considered homologous if they implement the same functional role and the region of similarity shared by them covers over 70% of each sequence. For further, comparative study of the genomes, the features of CD08_7 along with the other reference strains of *Nesterenkonia* were extracted from the RAST server on to an excel sheet and compared manually for presence of unique genes (presence of a gene homolog in strain CD08_7 but it’s absence in all other strains was considered as unique), potential pathogenicity determinants, genes involved in metabolic pathways related to virulence (comparative pathogenomics) and common genes among the strains as described previously (Nair et al., 2016).

## Results

### Characterization and Phylogenetic Analysis of Strain CD08_7

The strain designated as CD08_7 matched most of the phenotypic (cocci in shape, non-motile, negative for urease, indole and hydrogen sulfide production, negative for hydrolysis of gelatine and tween 80, nitrate reduction) and chemical [L-Lys-Gly-D-Asp peptidoglycan type, major menaquinones as MK-7 and MK-8 and major lipids as diphosphatidylglycerol (DPG), phosphatidylglycerol (PG), phosphatidyl inositol (PI)] characteristics of the genus *Nesterenkonia* and most of the features were matched with the species *N. jeotagli* (Yoon et al., 2006; negative for hydrogen sulfide and indole production, while positive for nitrate reduction, hydrolysis of starch, gelatine and tween 80 and for utilization of D-Glucose, D-Xylose, L-Arabinose, D-Cellobiose, D-Trehalose, Sucrose and Maltose; acid is produced from D-Galactose, D-Xylose and Mannitol, not from Lactose). 16S rRNA gene sequence showed that the strain CD08_7 correspond to the genus *Nesterenkonia* and is most closely related to *N. jeotgali* JG241^T^ (99.93% identity; 100% sequence completeness, one base difference of a total of 1444 bases) followed by *N. sandarakina* YIM 70009^T^ (99.79% identity: 99.8% sequence completeness, three bases difference of a total of 1459 bases), *N. halotolerans* YIM 7008^T^ (99.73% identity: 100% sequence completeness, four bases difference of a total of 1456 bases), *N. lutea* YIM 70081^T^ (99.38% identity :100% sequence completeness, nine bases difference of a total of 1456 bases) and *N. xinjiangensis* YIM 70097^T^ (97.05% identity:100% sequence completeness, 43 bases difference of a total of 1456 bases).

A combined phylogenetic tree (Neighbor joining, Maximum likelihood and Maximum parsimony) of strain CD08_7 was constructed using the 16S rRNA gene sequences of the closely related *Nesterenkonia* type strains and the reference strains NP1^T^, JCM 19054, F and AN1. Strain CD08_7 formed a separate branch with *N. jeotgali* JG-241^T^; likewise strain F clustered with *N. halobia* strain ATCC 21727^T^ and strain AN1 with *N. halotolerans* strain YIM 70084^T^ (**Figure 1**).

### Genome Features

The draft genome of strain CD08_7 consisted of 2,925,195 bp with G+C content of 67.6 mol%, 2531 predicted CDSs, 379 sub-systems and 52 RNAs. The final assembly contained eight contigs with *N*_50_ contig length of 731,296 bp and the largest contig assembled measured 813,259 bp. The genome size of *Nesterenkonia* sp. AN1 was the largest (3.0 Mb) among all genomes (ranging from 2.5 to 2.8 Mb). Highest genomic G+C content (71.4%) was of *Nesterenkonia* sp. strain F, followed by *Nesterenkonia* sp. strain AN1 (67.94%), *N. jeotgali* CD08_7 (67.6%), *N. massiliensis* strain NP1^T^ (63.0%), *Nesterenkonia* sp. strain JCM 19054 (61.1%). Other genome features of strain CD08_7 along with four reference genomes are shown in **Table 1**.

**Table 1:** Genome features of strain CD08_7 and other reference strain of genus *Nesterenkonia*.

Organism	*Nesterenkonia jeotgali* Strain CD08_7	*Nesterenkonia* sp. Strain AN1	*Nesterenkonia* sp. Strain F	*Nesterenkonia* sp. Strain JCM 19054	*Nesterenkonia massiliensis* Strain NP1^T^
Accession Number	LQBM00000000	JEMO00000000	AFRW00000000	BAXI00000000	CBLL00000000
Isolation source	Duodenal mucosa of CD patient	Salt Lake, Iran	Antarctic soil	Sea snail *Nassarius glans*	Feces of AIDS patient
Size (Mb)	2.9	3.0	2.8	2.5	2.6
Contigs	8	42	138	1086	175
Scaffolds	8	42	138	1086	19
G+C	67.6	67.4	71.5	67.1	62.9
tRNA	49	55	50	46	47
Other RNA	1	1	1	0	1
Number of RNAs	52	52	50	48	49
Number of subsystem	379	374	347	292	355
Coding sequences	2,531	2846	2480	3901	2435

### Identification of Virulence Determinants

Genome comparison of *N. jeotgali* strain CD08_7 was carried out among the other four reference strain of genus *Nesterenkonia.* The analysis revealed various categories of genes, among which, (1) virulence, disease and defense; (2) phages, prophages, transposable elements and plasmids; (3) stress response; (4) membrane transport and (5) iron acquisition, were further studied because of their extreme importance in contributing pathogenicity. A total of 213 genes were present in the five genomes analyzed had potential to confer pathogenicity. It was surprising to find that strain AN1 (an environmental isolate) had 174 pathogenicity genes. Strain CD08_7 had the second highest number of such determinants (163), strain F had 115, strain JCM 19054 had 123 and strain NP1 had 149. Only 59 of these determinants in strain CD08_7 were common in all the genomes, which shows the functional diversity among species of genus *Nesterenkonia*. Two genes of phage and prophage origin were unique in the genome of strain CD08_7.

### Virulence, Disease, and Defense

**FIGURE 2:**
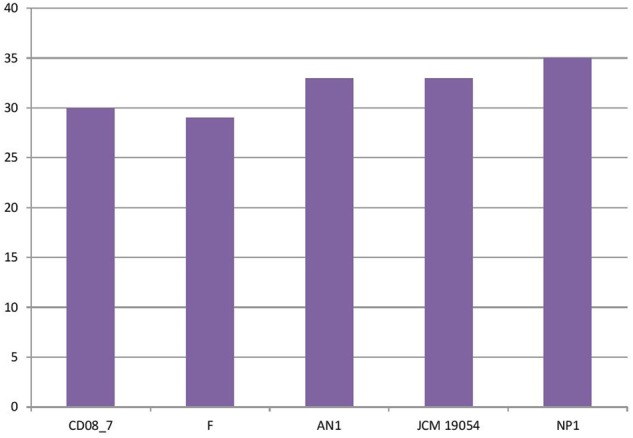
**Genes involved in virulence, disease, and defense**.

In this category around 30 genes were present in strain CD08_7, when compared to strain NP1^T^ which had 35, AN1 and JCM 19054^T^ had 33 followed by strain F which had 29 genes. Two further subcategories were studied under the category Virulence, disease and defense, for their probable contribution in pathogenesis: genes involved in resistance to antibiotics and toxic compounds and genes involved in invasion and intracellular resistance (**Figure 2**).

### Genes Involved in Resistance to Antibiotic and Toxic Compounds

**FIGURE 3:**
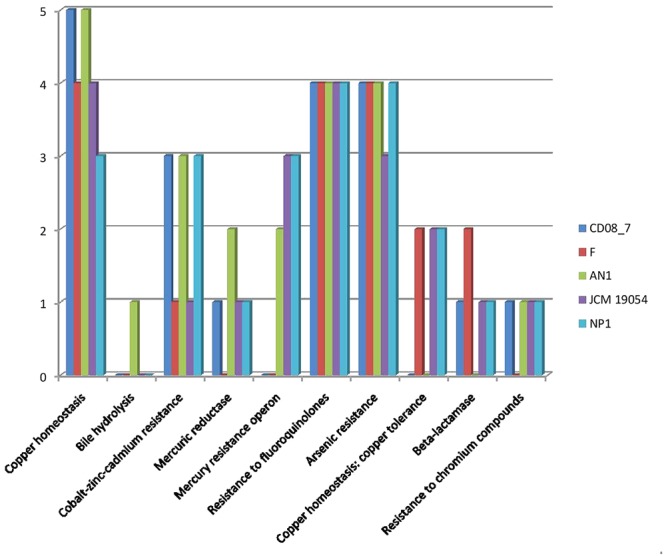
**Comparison of genes present in the *Nesterenkonia* strains involved in conferring resistance against antibiotics and toxic compounds**.

Resistance to antibiotics and toxic compounds is a primary feature which highlights the organisms as possibly virulent. There are a total of 29 genes present in this subcategory, 19 of which were present in strain CD08_7 whereas 22 in AN1 and NP1^T^. Strain JCM 19054 had 20 of these determinants in its genome, followed by Strain F that had 17 genes of these determinants in its genome for this subcategory (**Figure 3**). Strain CD08_7 is resistant to Nalidixic acid (10 μg) and sensitive to all other antibiotics of Icosa universal-2 panel, which is in accordance with the presence of mutant genes DNA gyrase subunit B (gyrB) and DNA gyrase subunit A (gyrA). Nalidixic acid inhibits the bacterial growth by damaging DNA replication process after blocking the action of above enzymes (Sugino et al., 1977). Mutations in these genes are supposed to cause resistance in microorganisms due to structural change in the active site of these enzymes.

### Genes Involved in Invasion and Intracellular Resistance

**FIGURE 4:**
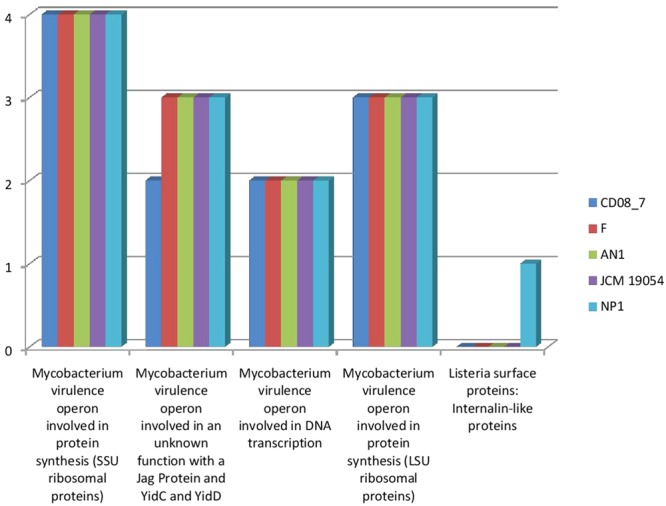
**Comparison of gene homologs present in the *Nesterenkonia* strains involved in invasion and intra cellular resistance**.

*Mycobacterium* has SSU and LSU ribosomal proteins which contribute in the development of tuberculosis (Nair et al., 2016). This category highlights the presence of these determinants described in *Mycobacterium* as a characteristic for invading the host and disease progression. Four subsystems were present in strain CD08_7, strain AN1, strain JCM 19054, and strain F whereas strain NP1^T^ had one gene of an additional subsystem. These results show that strain CD08_7 has the potential to be invasive (**Figure 4**).

### Genes Involved in Membrane Transport

**FIGURE 5:**
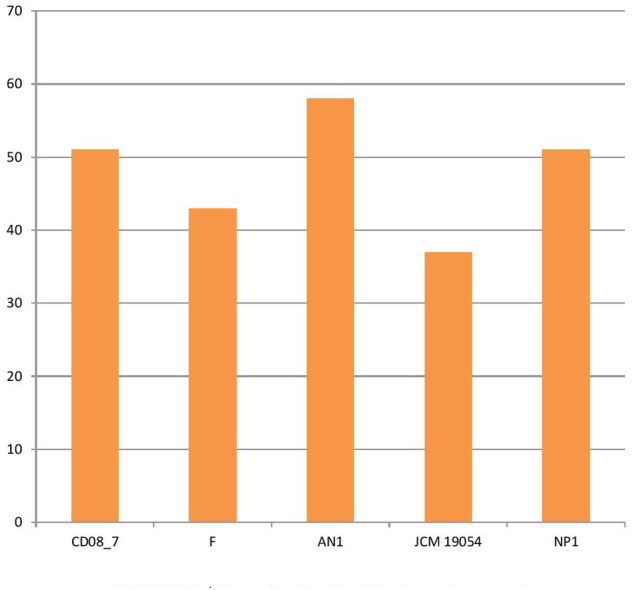
**Genes involved in Membrane transport**.

Several pathogenic bacteria have evolved mechanisms to counteract the host mediated degradation via endocytic pathway, modulating the membrane transport proteins is one such mechanism through which the bacteria are able to use a T3SS (type III secretion system) or T4SS (type IV secretion system) to translocate their proteins across the membrane, where they manipulate the host proteins residing in the cytoplasm. Other bacteria have developed eﬄux pumps to keep intracellular concentrations of antibiotics low (Alix et al., 2011). Strain AN1 had 58 transport genes in its genome, followed by strains CD08_7 and NP1^T^ with 51, strain F with 43 and strain JCM 19054 with 37. Strain AN1 had some additional transport genes of subcategories, protein secretion system type II (3), ABC transporters (2), TRAP transporters (3) and copper transporters (1) that were absent in CD08_7 (**Figure 5**).

### Stress Response

**FIGURE 6:**
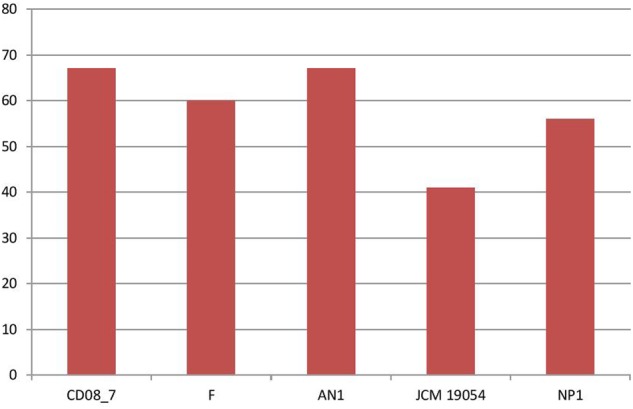
**Genes involved in Stress response**.

Stress response is activated in bacteria when they encounter unfavorable conditions in the host environment. The response mechanism involves adaptation to new conditions by regulation of several molecular pathways that control transcription, translation and post-translation modifications (Rukhsana et al., 1996). Therefore, bacteria having stress response genes in their genomes are able to cope with the oxidative stress produced by human immune cells and osmotic stress due to host osmolytes (Rothe et al., 2012; Paiva and Bozza, 2014). In this category 67 genes were present in strain CD08_7 and AN1, while strain F had 60, strain NP1^T^ had 56 and JCM 19054 had 41 (**Figure 6**).

#### Osmotic Stress Genes

**FIGURE 7:**
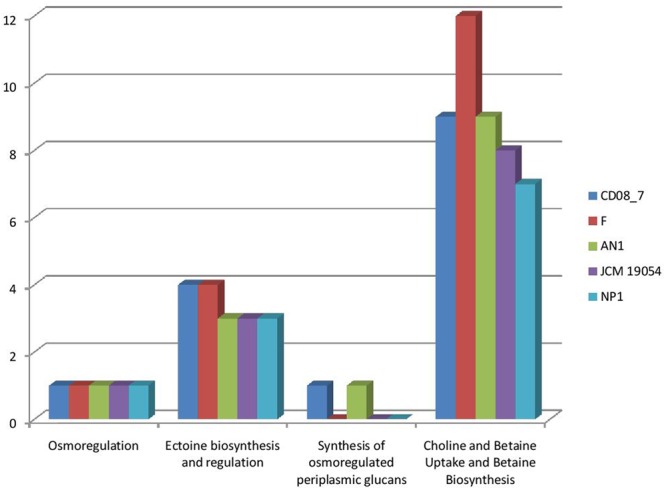
**Comparison of genes present in the *Nesterenkonia* strains involved in Osmotic Stress**.

Osmotic stress is encountered by pathogenic bacteria due to change in the osmolarity while facing adverse osmolyte concentrations. Microbes inhabiting the human intestine face diverse types of osmotic stress exerted by different osmolytes, i.e., carbohydrates, sugars, proteins or fiber components, that can be counteracted by the genes of osmoregulation, if a bacterium contains them (Lucht and Bremer, 1994; Sleator and Hill, 2002; Rothe et al., 2012). Strain CD08_7 possesses four sub-systems which may aid it in osmoregulation when facing stress conditions. Similarly four sub-systems were present in strain AN1 and 3 in strain NP1^T^, JCM 19054 and strain F (**Figure 7**). Such osmostress responsive systems contribute to the virulence potential of several pathogenic bacteria. The conclusion can be drawn from the above results that strain CD08_7 possesses the potential to sustain osmotic stress in the host.

#### Oxidative Stress Genes

**FIGURE 8:**
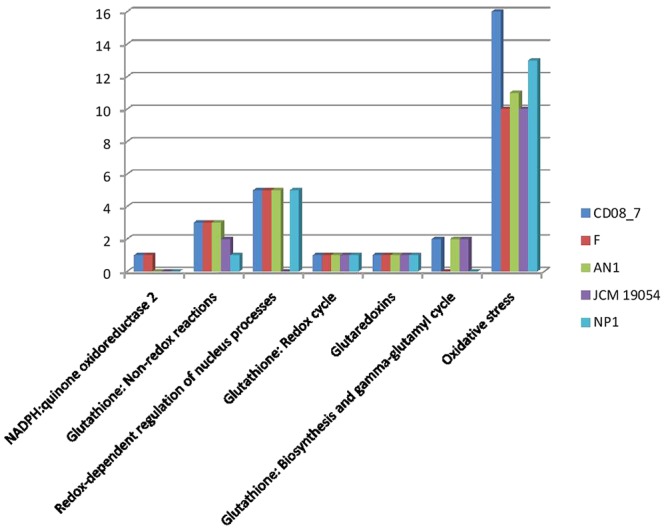
**Comparison of genes present in the *Nesterenkonia* strains involved in Oxidative Stress**.

The intestinal barrier function of CD patients is generally dysregulated (Uhde et al., 2014), thus intestinal microbes are expected to get more exposure to phagocytes and other immune cells of the intestinal mucosa. As a defense mechanism against microbes, dendritic cells and B-lymphocytes produce reactive oxygen species (ROS) that cause oxidative stress to inhabiting intestinal microbes (Paiva and Bozza, 2014; Cachat et al., 2015; Stoiber et al., 2015). Interestingly, there are evidences of increased ROS generation at duodenal mucosa of CD patients (Murray et al., 2002; Daniels et al., 2005). Strain CD08_7 contains a total 16 genes which may help it to cope with the oxygen stress in the host environment. Strain NP1^T^ had 13 such genes followed by AN1 which had 11 while strains F and JCM 19054 had 10 (**Figure 8**).

### Genes of Phages, Prophages, Transposable Elements and Plasmids

**FIGURE 9:**
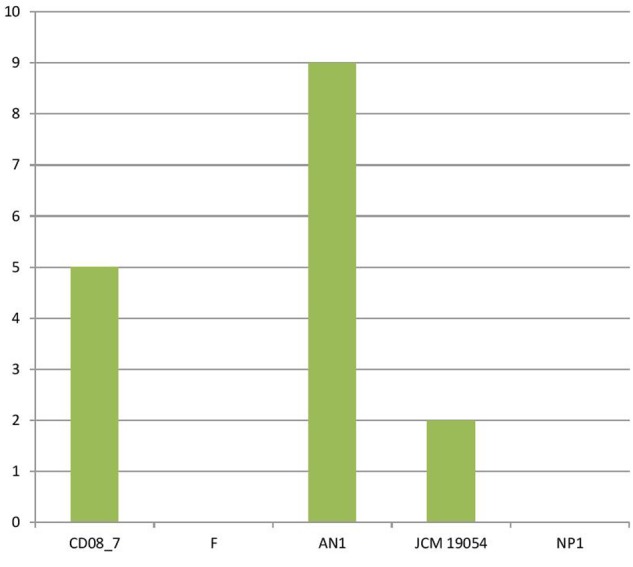
**Genes involved in Phages, Prophages, Transposable elements, Plasmids**.

**FIGURE 10:**
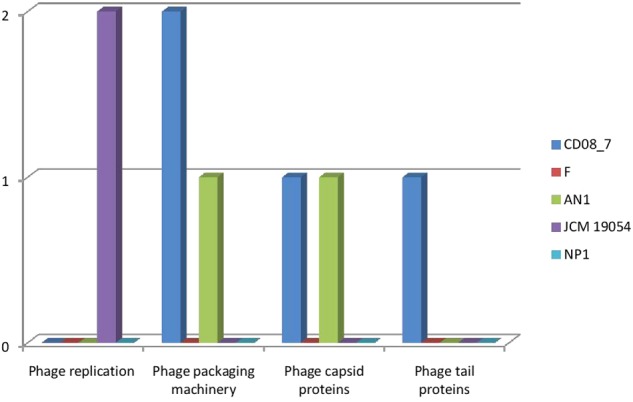
**Comparison of genes present in the *Nesterenkonia* strains involved in Phages, Prophages**.

The genes encoding phages, transposable elements and prophages contribute to variability and thus pathogenic success by promoting recombination in bacterial genomes. Strain AN1 contains nine genes in this category, thus strain CD08_7 was second with five followed by strain JCM 19054 which had only two and strains F and NP1^T^ had none (**Figure 9**). Although strain CD08_7 had the highest number of genes (4) in sub-category phages and prophages, it lacked genes for phage replication. The only other strain possessing phage-related genes (just two for replication) was JCM 19054 (**Figure 10**).

### Genes for Iron Acquisition and Metabolism

**FIGURE 11:**
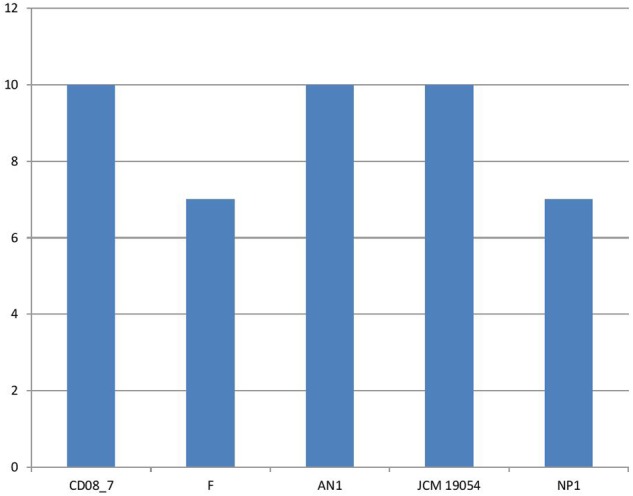
**Genes involved in iron acquisition and metabolism**.

**FIGURE 12:**
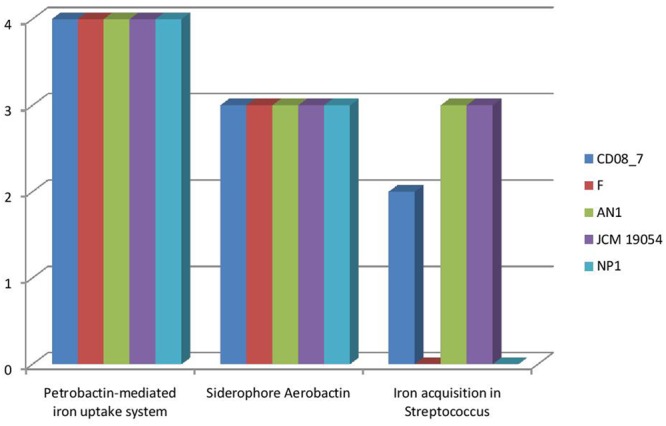
**Comparison of genes present in the *Nesterenkonia* strains involved in siderophores and iron acquisition and metabolism**.

It has been shown that iron is essential for growth in bacteria. Within the host bacteria and host cells compete for iron. Bacteria have developed strong mechanisms to capture iron from the host like receptors for eukaryotic iron binding proteins and siderophores, which have high affinity for iron. Therefore, iron acquisition capability is considered as an important measure of pathogenicity associated with any bacterial strain (Baron, 1996). Among the strains analyzed, CD08_7 comprises the highest number of genes related to iron acquisition (10) like strains AN1 and JCM 19054, whereas strains NP1^T^ and F had 7 genes (**Figure 11**). Some of the most important genes in this category were homologs of *Streptococcus* Siderophore Aerobactin, Petrobactin-mediated iron uptake system and iron acquisition (**Figure 12**).

## Discussion

The present study analyzed the genome sequence of clinically isolated *N. jeotgali* strain CD08_7 and revealed several features that highlight it as a probable pathogen. This clinical strain of *Nesterenkonia* possesses as genetic makeup different from those of the non-clinical strains used here for comparison: it contains higher numbers of genes for virulence (iron acquisition systems, antibiotic resistance, multi drug resistance, oxidative stress, and osmotic stress resistance) suggesting adaptation to a host-associated lifecycle. Most of the *Nesterenkonia* strains described to date are of non-clinical origin and many putative virulence genes have been found in strain CD08_7. This fact suggests that this *Nesterenkonia* strain is adapted to live in the human intestine and may be relevant in the CD. This study has provided new insights into the pathogenomics of strain CD08_7 which strengthen our suspicion that this strain may be a pathogen associated to CD. We hope that these data will prove beneficial in furthering our understanding of the clinical pathology of this disease.

## Future Work

This study highlights the attributes of virulence in strain CD08_7 as described by annotated genome sequences and its comparative pathogenomics analysis with reference strains within the same genus. A well planned study focused on determining the prevalence of *Nesterenkonia* spp./*N. jeotgali* in duodenal mucosa of CD and control subjects and its association with disease activity markers and symptoms is still pending. Such a study will allow us to understand the role of this organism and its contribution in the development of CD.

## Nucleotide Sequence Accession Number

The *Nesterenkonia jeotgali* strain CD08_7 whole genome shot gun project has been deposited at DDBJ/EMBL/GenBank under project accession number LQBM00000000 locus LQBM01000000 and comprises sequences LQBM01000001-LQBM01000008.

## Ethics Statement

The study protocol was approved by the Institute’s Ethics Committee of Postgraduate Institute of Medical Research and Education, Chandigarh, India. An informed written consent was obtained from the participant. CD was diagnosed based on serum IgA anti-tissue transglutaminase antibody [tTG-Ab] and duodenal biopsy suggestive of villous atrophy (Oberhuber et al., 1999).

## Author Contributions

Performed experiments: RN, AC, and GK; RK provided surgical samples; Planned and executed experiments analyzed data and wrote manuscript: DD, SB, and SM. All authors read and approved the final manuscript.

## Conflict of Interest Statement

The authors declare that the research was conducted in the absence of any commercial or financial relationships that could be construed as a potential conflict of interest.

## Footnotes

1 http://www.ccamp.res.in/

2 http://rast.nmpdr.org/

3 http://www-is.biotoul.fr/
